# Bioinspired iterative synthesis of polyketides

**DOI:** 10.3389/fchem.2015.00032

**Published:** 2015-05-21

**Authors:** Kuan Zheng, Changmin Xie, Ran Hong

**Affiliations:** CAS Key Laboratory of Synthetic Chemistry of Natural Substances, Shanghai Institute of Organic Chemistry, Chinese Academy of SciencesShanghai, China

**Keywords:** polyketides, iterative synthesis, bioinspired synthesis, polyol, biosynthesis

## Abstract

Diverse array of biopolymers and second metabolites (particularly polyketide natural products) has been manufactured in nature through an enzymatic iterative assembly of simple building blocks. Inspired by this strategy, molecules with inherent modularity can be efficiently synthesized by repeated succession of similar reaction sequences. This privileged strategy has been widely adopted in synthetic supramolecular chemistry. Its value also has been reorganized in natural product synthesis. A brief overview of this approach is given with a particular emphasis on the total synthesis of polyol-embedded polyketides, a class of vastly diverse structures and biologically significant natural products. This viewpoint also illustrates the limits of known individual modules in terms of diastereoselectivity and enantioselectivity. More efficient and practical iterative strategies are anticipated to emerge in the future development.

Polyketides are a class of secondary metabolites being impressive not only with structurally intriguing carbon skeletons but also with strong pharmacological relevance. A wealth of biologically important activities including antitumor, antibiotic, cytostatic, antiparasitic and immunosuppressive properties, and many of them or their derivatives have become therapeutics for clinical use. Over the past five decades tremendous progress has been advanced in the field of chemical synthesis of polyketides including macrolides, polyphenols, polyethers, polyenes, and enediynes. Numerous innovative strategies and tactics have been pursued and illustrated in many elegant total syntheses. However, polyketide with a moderate–level complexity still posts a formidable challenge to cumulate enough quantity for clinical evaluation. The success of anticancer drug Halaven® (Eribulin) from Eisai and the Kishi's lab is considered as a triumph in the field of polyketide synthetic chemistry. Nevertheless, how many can polyketide drug leads be eventually evolved to clinical use from the synthetic laboratory? Sophisticated approaches to rapidly and flexibly access the stereoarrays are still highly demanded from the synthesis prospect.

It is informative when we closely analyze how Nature forms biomolecules in an iterative strategy by enzymatic assembly of simple building blocks. Inspired by this powerful strategy, chemists have developed similar assembly–line processes to synthesize biopolymers (such as polypeptides, oligonucleotides, and oligosaccharides) (Caruthers, 1985; Merrifield, 1985; Seeberger and Haase, 2000) and other supramolecular systems such as dendrimers, cyclacenes, oligophenylenes, and polyspiranes. This methodology becomes privileged in synthetic supramolecular chemistry (Feuerbacher and Vögtle, 1998). However, it is still undervalued in natural product synthesis, particularly in the field of polyketides. In this Review, we highlight a number of iterative total syntheses of some important classes of polyketide such as polyenes, skipped polyols and polypropionates. These examples are not meant to be comprehensive since many elegant syntheses are not included.

It is now wildly accepted that the polyketides synthases (PKS), which are similar to fatty acid synthases (FAS), are responsible for the biogenicity of polyketides (Katz, 1997; Staunton and Weissman, 2001). The key carbon–carbon bond formation in chain propagation is realized by repetitive decarboxylative Claisen condensation of thioester powered by PKS. Acid derivatives such as acetyl–CoA, malonyl-CoA, and methylmalonyl-CoA are employed as simple building blocks in chain elongation. A series of functional units or modules are ordered in sequence in the PKS, and each of the module contains several domains with different functions (Figure 1A). The modules are arranged in an ordered way that polyketides can be assembled iteratively and efficiently (Dutta et al., 2014; Whicher et al., 2014). Generally, at least 3 domains [ketosynthase (KS), acyltransferase (AT), and acyl carrier protein (ACP)], are required for one iterative cycle of the chain extension. Other domains include ketoreductase (KR), dehydratase (DH) and enoyl reductase (ER).

**Figure 1:**
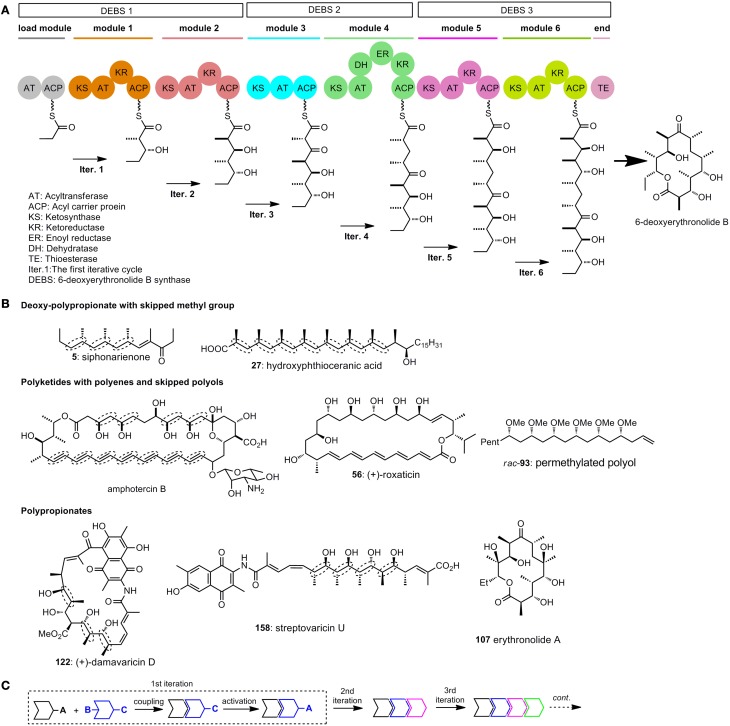
**(A)** Model for the domain organization of 6-deoxyerythronolide B synthase (Katz, 1997); **(B)** Selected polyketides with repetitive structure units. Elegant iterative synthesis of the deoxy-polypropionate, polyenes, skipped polys, and polypionates will be introduced in the following test; **(C)** A general strategy for iterative synthesis.

To illustrate the function of each module, we draw a model in which stepwise synthesis of 6-deoxyerythronolide B are showed (Staunton and Weissman, 2001) (Figure 1A). The PKS for biosynthesis of 6-deoxyerythronolide B (DEBS) are multienzyme polypeptides which consist of three synthases DEBS 1, 2, and 3. The load module in DEBS 1 have two domains, AT and ACP. These two domains indicate the beginning of polyketide assembly. The KR domain, as shown in the first iterative cycle (iter. 1), has the function of reducing ketones to alcohols. Module 3 does not contain the KR domain, so ketone group survives in iter. 3. DH has the function of forming double bonds, while ER will reduce the double bonds to saturated ones. The addition of DH and ER domains to a basic extending module (KR + AT + ACP) will give a saturate extension moiety, as shown in iter. 4. By altering the domains between AT and ACP in each module, different functional groups such as ketones, alcohols and alkenes can be arranged in the polyketide in an organized way.

The repetitive module 1 in a PKS will give the polypropionates, the repetitive module 4 in a PKS is expected to generate deoxy-polypropionates, while repetitive module 4 without ER domain in a PKS will provide polyenes. Polyketides are endowed with intrinsic repeatability through the enzyme catalyzed iterative synthesis (Figure 1B). As a imitation to nature, iterative sequences have already been used by many chemists to synthesize polyketides.

In a general illustration of monodirectional iterative sequence (Figure 1C), two different building blocks can either be utilized in the key linking reaction, between two of which the protected sites (such as **B** and **C** in Figure 1C) are activated. After a few iterations large arrays of targets with defined structures can be obtained in a straightforward manner.

Compared with other customized strategies, the whole synthetic sequence becomes much simpler and more flexible, thus holding the potential for automated production by non–specialists. In the context of polyketide total synthesis, the past decades have witnessed the development of several catalytic and non-catalytic processes where such streamlined construction of stereo-defined polyketide arrays can be realized. Two elegant reviews on iterative strategies focusing on deoxypropionates with skipped methyl groups have appeared (Hanessian et al., 2006; Horst et al., 2010).

## Deoxy-polypropionate with skipped methyl groups

**Figure 2:**
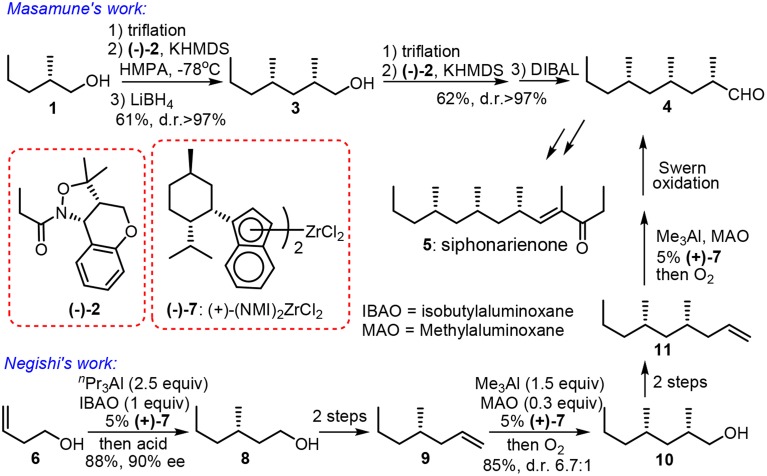
**Synthesis of siphonarienone (Abiko and Masamune, 1996; Magnin-Lachaux et al., 2004)**.

**Figure 3:**
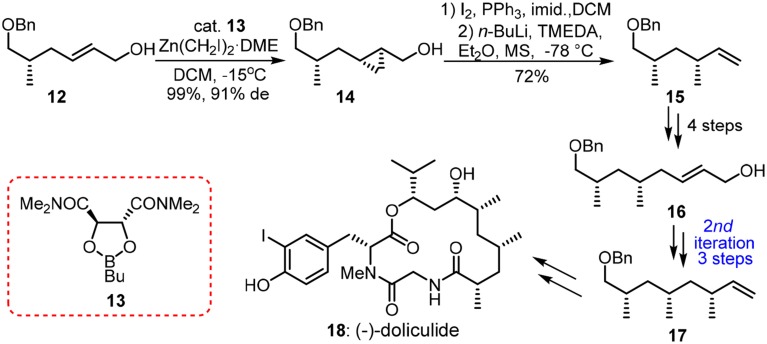
**Synthesis of (−)-doliculide (Ghosh and Liu, 2001)**.

When methylmalonate units are incorporated into the polyketide chain and the resulting β-keto functionalities undergo fully enzymatic reductions and dehydrations, the skipped methyl carbon chain is formed. Pedant methyl groups in naturally derived deoxypropionate have a bias for all-*syn* orientation in order to alleviate the number of destabilizing interactions during the chain-elongation process (Hoffmann, 2000). Abiko and Masamune (1996) have employed a chiral auxiliary-based asymmetric alkylation in the first synthesis of (+)-siphonarienone (**5**, Figure 2). The potassium enolate of benzopyrano-isoxazolidine **2** reacted with the triflate of β-branched alcohol **1** (obtained via resolution) with good diastereoselectivity. The reductive removal of the chiral auxiliary is needed to set the next iterative alkylation. Another noteworthy approach toward **5** was reported via the Negishi Zr-catalyzed asymmetric carboalumination (ZACA) (Magnin-Lachaux et al., 2004). Staring from homoallylic alcohol **6**, the ZACA protocol introduced an ω-*n*-propyl group in the presence of chiral zirconocene derivative **7**. Three iterative carboaluminations furnished an all *syn*-product **4** with high diastereoselectivity while those minor diastereomers were readily depleted by column chromatography. The total synthesis of the potent antitumor agent (−)-doliculide (**18**) by Ghosh and Liu (2001) disclosed a repetitive cyclopropanation-fragmentation (Figure 3). The synthesis of its polyketide unit **17** commenced with the Charette asymmetric cyclopropanation of allylic alcodol **12** by dioxaborolane **13**. Subsequent iodination and lithium-holagen exchange-initiated ring-opening delivered alkene **15**. A four-step sequence is further required to deliver a homologated allylic alcohol **16** for next iteration.

**Figure 4:**
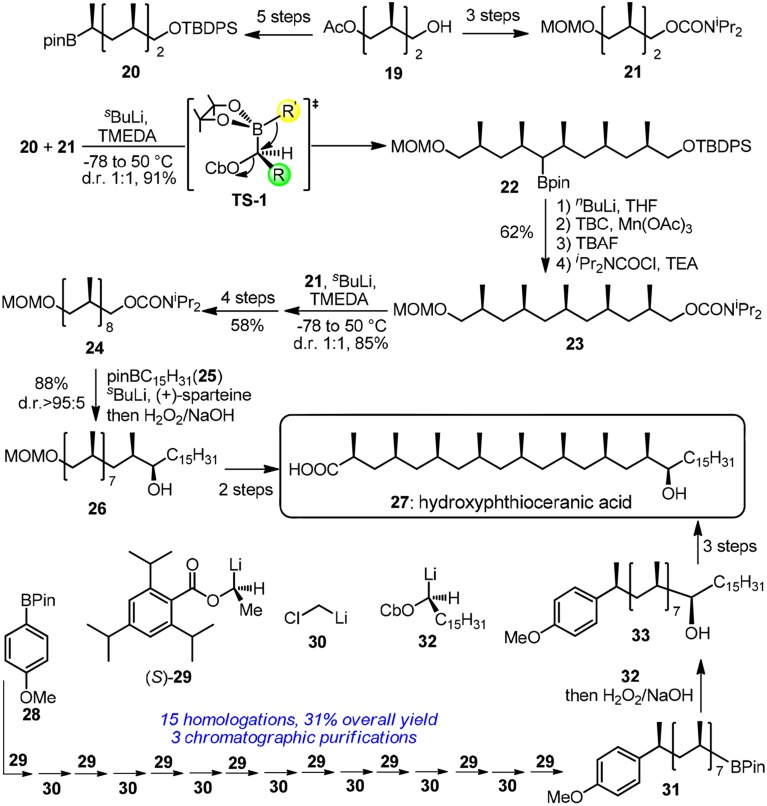
**Synthesis of hydroxyphthioceranic acid (Rasappan and Aggarwal, 2014; and Balieu et al., 2015)**.

Hydroxyphthioceranic acid (**27**, Figure 4) is a constituent of the cell-wall lipid of pathogenic germ *Mycobacterium tuberculosis*. Pioneering work in iterative construction of its all-*syn* skipped methyl deoxypropionate array was dictated by Minnaard and Feringa group via catalytic iterative 1,4-addition (Geerdink et al., 2013). Aggarwal and co-workers have developed a traceless iterative strategy based on the Matteson rearrangement (Matteson, 2013; Rasappan and Aggarwal, 2014). Bifunctional building blocks **20** and **21** can be synthesized in several steps from the commercially available alcohol **19**. Homologated boronic ester **22** was obtained via a stereospecific 1,2-migration reaction in **TS-1**. Treatment of α-lithiated **22** with 4-*tert*-butylcatechol/Mn(OAc)_3_·2H_2_O and subsequent carbamoylation furnished the protodeboronated product **23**. Next round of lithiation-borylation-protodeboronation incorporating **21** was found to be challenging and MeLi should be used instead of *n*BuLi to form a more stereo–demanding boronate complex. Undoubtedly, a more reliable protodeboronation protocol which is especially suitable for bulky alkyl pinacol boronic esters is still in request. More resonates with Hendrickson's ideal synthesis, the same group has revised an assembly-line strategy toward **27** (Balieu et al., 2015), where 16 staggered homologations are sequentially required. The required methylene units were inserted via boronic ester homologations with α-chloromethyllithium **30**. Other homologations were dominated by chiral lithiated building blocks **29** and **32**. Aggarwal's lithiation-borylation method can be also used in the stereoselective synthesis of other challenging targets (Leonori and Aggarwal, 2014), such as alkyl chain with adjacent acyclic quaternary-tertiary motifs (Blair et al., 2014) or alkyl chain with ten contiguous methyl groups (Burns et al., 2014).

## Polyene-type polyketides

In biosynthetic machinery leads to polyene-type polyketides, enoyl reductase (ER) takes no active part after each β-keto reduction and dehydration in several consecutive chain elongation processes, thus providing natural products with considerable structural unsaturation. Synthetic approaches to such conjugated double bond frameworks are made challenging by their sensitivities to oxygen, light and acidic conditions, as well as the issues concerning good stereocontrol. Controlled iterative cross coupling reactions play an important role in the construction of conjugated organic compounds (Wang and Glorius, 2008). Very recently, Burke and co-workers have devised a versatile iterative bifunctional building block-based cross-coupling strategy for accessing polyene natural products such as fatty acids, polyketides, hydrid peptide/polyketides and polyterpines (Gillis and Burke, 2007, 2009). The instability of alkenyl boronic acid has limited its usage in the synthesis. In Burke's *N*-methyliminodiacetic acid (MIDA) protected boronic acid, the hybrid orbital of boron changes from *sp*^2^ to *sp*^3^. This has greatly increased the stability of MIDA borates and attenuated the reactivity of boronic acid. Oligomerization is inhibited when bifunctional MIDA borates are used in iterative cross coupling. Meanwhile, the MIDA protecting group can be easily removed under basic aqueous conditions. Currently, over 170 MIDA building blocks are commercially available from *Sigma-Aldrich* (Woerly et al., 2014).

**Figure 5:**
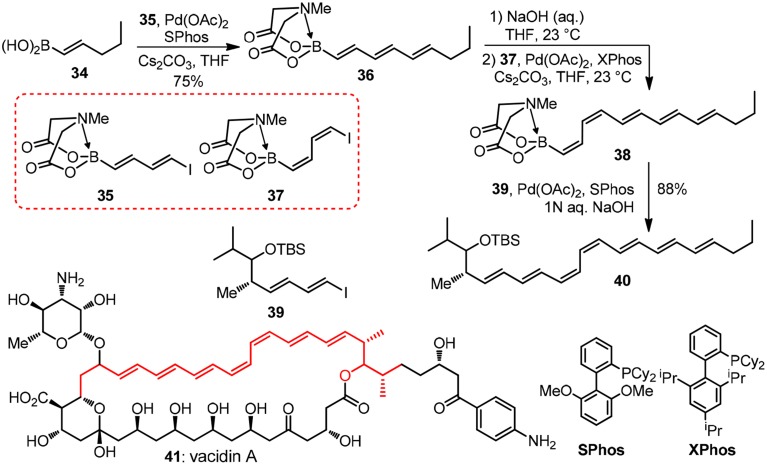
**Synthesis of the heptaene fragment of vacidin A (Lee et al., 2010)**.

Taking their modular synthesis of the heptaene core of vacidin A as an illustration (**41**, Figure 5). In the structure of which a problematic *cis*-diene is embedded (Lee et al., 2010), the target **41** as well as other polyene macrolides such as amphotericin B and nystatin, are found to display an unique mode of action by forming ion channels when dealing with systematic fungal infections. Bornic aicd **34** couples with vinyl iodide moiety of bifunctional building block **35** to form **36** successfully under the palladium catalyzed conditions without oligomerization **35**. The MIDA protecting group in **36** can be easily unmasked with NaOH aqueous solution and subsequent coupling reaction of the protecting group removed boronic acid with **37** will generate the challenging cis-diene moiety in **38**. The last iterative Suzuki-Miyaura coupling reaction of **38** and building block **39** will form the polyene moiety of vacidin A. By uniting a collection of MIDA building blocks, a vast number of complex polyene motifs can be made with excellent stereocontrolled iterative cross-coupling reaction. Recently, great achievements have been made by Burke group (Li et al., 2015) in the field automated process. The authors have synthesized 14 distinct classes of small molecules using this automated process. Interestingly, the challenging purification problem is solved by using MIDA borates' unusual binary affinity for silica gel with certain pairs of eluents. The MIDA borates show minimal mobility on thin-layer silica gel chromatography when eluting with MeOH/Et_2_O, while they are rapidly eluted when THF is used.

## Polyketides with skipped polyols

**Figure 6:**
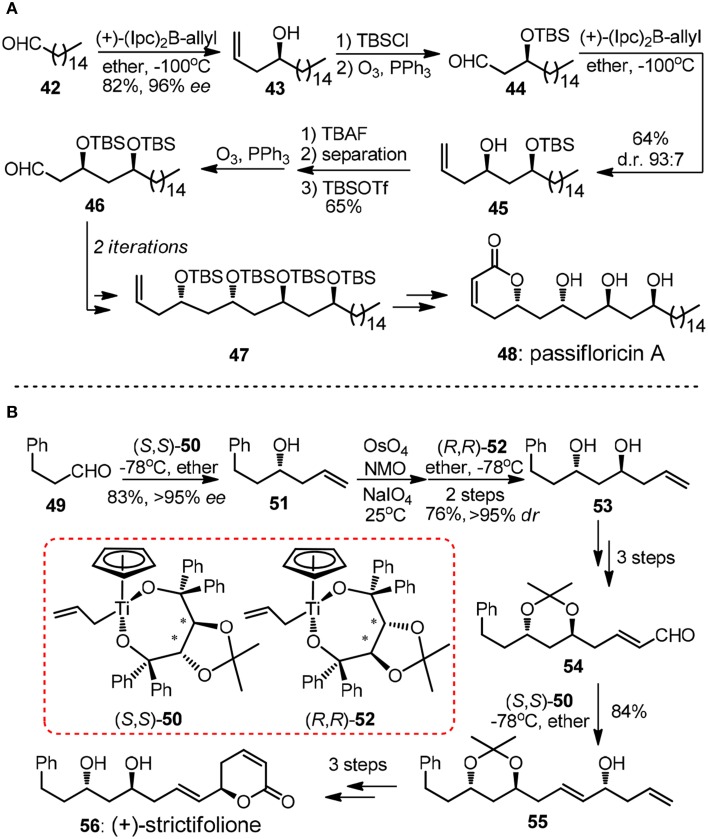
**(A)** Synthesis of published structure of passifloricin A (Garcia-Fortanet et al., 2003); **(B)** Synthesis of (+)-strictifolione (BouzBouz and Cossy, 2003).

As one of the key structural elements, polyacetate-derived 1,3-polyol unit is featured in vast collection of polyketide natural products. During the past decades, numerous synthetic methodologies have been advanced to stereoselectively introduce 1,3-diols or higher order 1,3-polyol arrays (Bode et al., 2006). Methods based on allylation of carbonyl groups are frequently encountered in literature. The repetitive allylmetallation of aldehydes is of great interest since typically only three steps including allylation, protection and oxidative aldehyde regeneration are required in a single iteration. An illustrative example of Brown's asymmetric allylation is showed in Marco's synthesis of α-pyrone natural product passifloricin A (**48**, Figure 6A) (Garcia-Fortanet et al., 2003). After the first iteration, the resulting β-silyoxy aldehyde **46** was subjected to second allylation to yield homoallylic alcohol **45**.

The undesired diastereomer of **45** could only be removed as the corresponding diol after desilylation by column chromatography, thus addition steps were required in order to afford the product in a high optical purity. Apart from chiral allylborane, Duthaler-Hafner reagent **50** and **52** were nicely implemented in repetitive synthetic operations during the concise synthesis of antifungal α-pyrone (+)-strictifolione (**56**, Figure 6B) (BouzBouz and Cossy, 2003). It is notable that 1,3-*anti* diol **53** can be produced via direct allylation of the intermediacy β-hydroxyl aldehyde in excellent diastereoselectivity.

**Figure 7:**
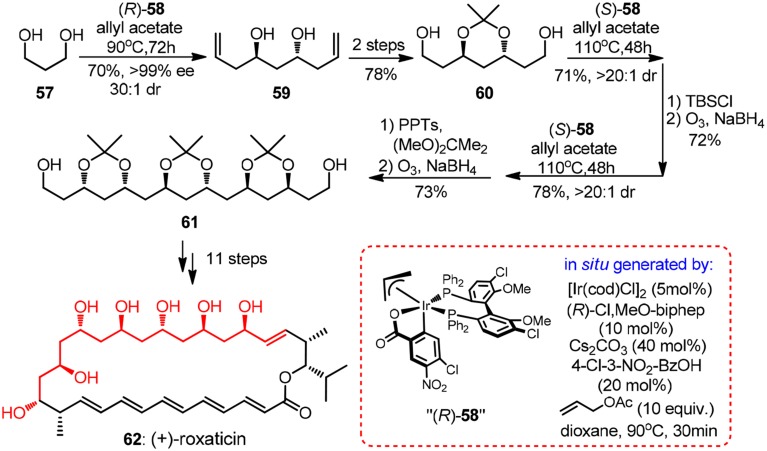
**Synthesis of (+)-roxaticin (Han et al., 2010)**.

Evidently, it is of immense potential to conduct allylation in a catalytic version so as to preclude the usage of stoichiometric chiral reagent. An intriguing chiral sulfonamide-catalyzed allylchromation protocol was described by Kishi group (Zhang et al., 2008). In the same year, Krische and co-workers showcased a breakthrough Ir-catalyzed transfer hydrogenation allylation reaction (Kim et al., 2008). Homoallylic alcohols of high optical purity can be obtained using a modified chiral iridium *C*,*O*-benzoate complex **58**. Oxo-pentaene macrolide (+)-roxaticin (**62**, Figure 7) constitute an appealing target due to its strict polyacetate origin. An impressing short synthesis of **62** by Krische group relies principally on an iterative two-dimensional assembly of 1,3-diol **57**, which can be considered as unstable malondialdehyde equivalent (Han et al., 2010). The first allylation can be conducted on a large scale using a less costly (*R*)-BINAP analog to yield *C*_2_-symmetric diol **59** with same excellent levels of stereocontrol, albeit in moderate isolating yield. It is quite remarkable that in each dimensional allylation, the stereochemical bias of the *in situ* generated iridium catalyst overrides the intrinsic facial selectivity of the transient chiral β-branched aldehydes. The acetonide-protected polyacetate fragment **61** processing six chiral centers was prepared in an elegant nine-step sequence.

**Figure 8:**
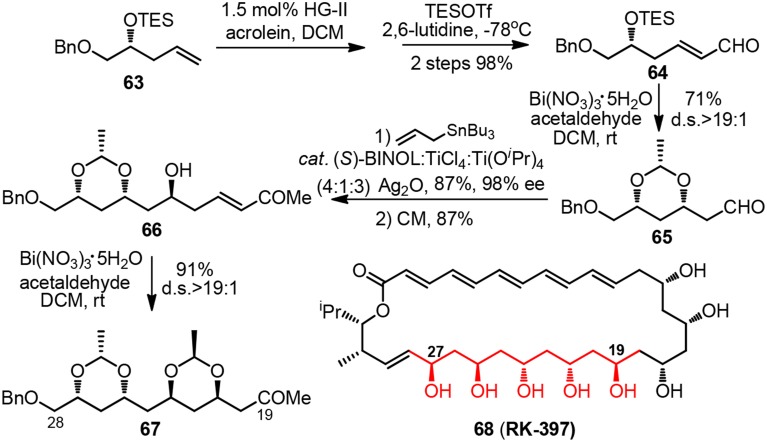
**Synthesis of the C18-C28 polyol fragment of RK-397 (Evans et al., 2012)**.

Synthetic pursuit of the C19-C28 polyacetate unit of macrolide RK-397 (**68**, Figure 8) was initiated by Evans and co-workers to demonstrate the convenience of their newly developed bismuth-mediated protocol for diastereoselective formation *syn*-1,3-dioxanes (Evans et al., 2012). Treatment of chiral δ-trialkylsilyloxy α,β-unsaturated aldehyde **64** or δ-hydroxy α,β-unsaturated ketone **66** with excessive amount of acetaldehyde and bismuth(III) nitrate as a substoichiometric mediator furnished ethylidene acetal-protected *syn*-1,3-dioxane **65** or **67** in good yield. The reaction proceeds via a thermodynamically controlled hemiacetal/oxa-conjugate addition process, and gives rise to product bearing electron-withdrawing group which can be directly utilized in consecutive Keck-Maruoka allylation.

**Figure 9:**
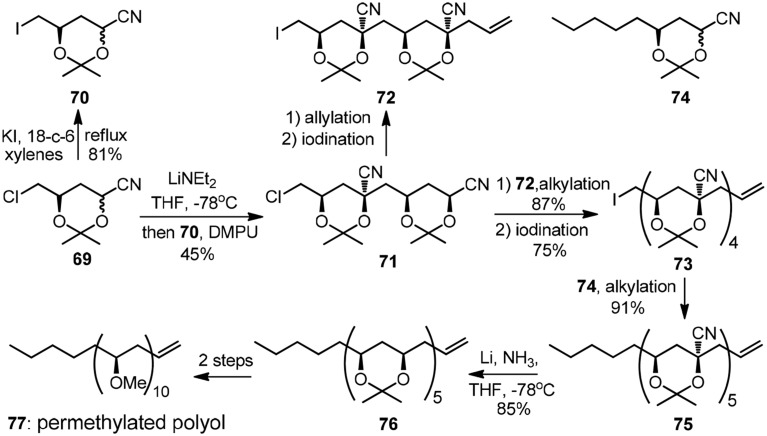
**Synthesis of the permethylated polyol (Rychnovsky and Griesgraber, 1992)**.

In analogy to Krische's two-directional modular synthesis of (+)-roxaticin, the rapid growth of alternating polyol segment can also be achieved by iterative stereoselctive alkylation of cyanohydrin acetonides. As was exemplified by Rychnovsky's early convergent synthesis of isotactic permethylated polyol (**77**, Figure 9) (Rychnovsky and Griesgraber, 1992), the alkylation of **69** with its electrophilic-activated derivative **70** gave a single isomer **71** owing to a kinetic anometric effect. A secondary alkylation followed by electrophilic activation delivered **73**, which was subjected to the final alkylation with cyanohydrin acetonide **74** to yield pentanitrile **75**. The featured reductive removal of the five axial cyano groups was realized by dissolving metal reduction with complete retention of configuration.

**Figure 10:**
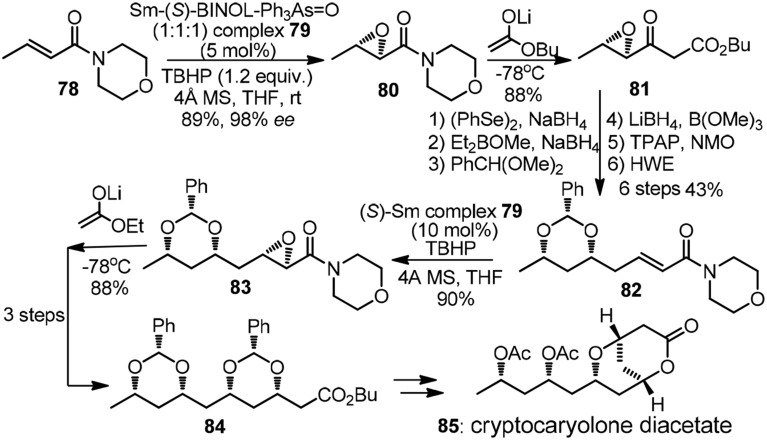
**Synthesis of cryptocaryolone diacetate (Tosaki et al., 2004)**.

The pioneering studies on iterative 1,3,5-triols synthesis based on asymmetric epoxidation followed by reductive epoxide ring-opening was reported by the group of Sharpless (Katsuki et al., 1982). The chain extension protocol was modified by Nicolaou et al. (1987) and further applied in their total synthesis of amphotericin B. The Shibasaki group, on the other hand, established a new catalyst-controlled epoxidation promoted by complex **79** (Tosaki et al., 2004). All possible isomers of 1,3,5,7-tetraol array were readily accessible with equally high efficiency. Synthetic endeavor on cryptocaryolone diacetate (**85**, Figure 10) was presented to showcase its versatility. α,β-Epoxy morpholinyl amide **80** was subjected to Claisen-type condensation to form β-keto ester **81**, the later of which underwent subsequent 6 steps to complete one iteration. Yadav et al. (2007) also demonstrated a chiral pool strategy toward **85** by means of consecutive use of Prins cyclisation followed by reductive tetrahydropyran ring opening.

**Figure 11:**
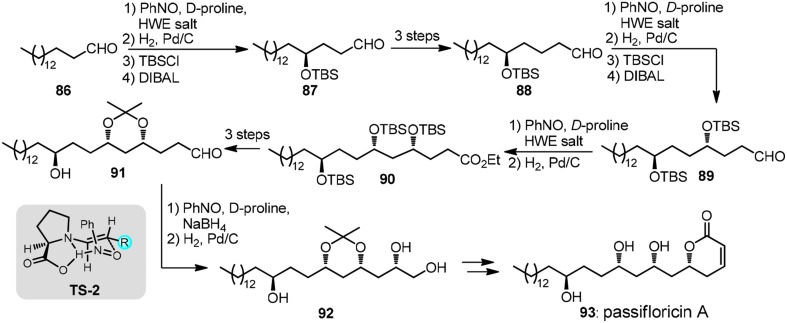
**Synthesis of passifloricin A (Kumar et al., 2012)**.

An iterative organocatalytic approach was recently displayed by Kumar et al. (2012) in their total synthesis of revised structure of passifloricin A (**93**, Figure 11). In typical iteration, palmitaldehyde **86**, upon sequential treatment of nitrosobenzene and *D*-proline followed by Horner-Wadsworth-Emmons (HWE) olefination was transformed into enantioenriched *O*-amino allylic alcohol which was hydrogenated to provide δ-hydroxy ester. *C*_2_-homologated aldehyde **87** was afforded after silylation and DIBAL-H reduction. The stereochemical outcome of α-aminoxylation can be rationalized via **TS**-**2**.

**Figure 12:**
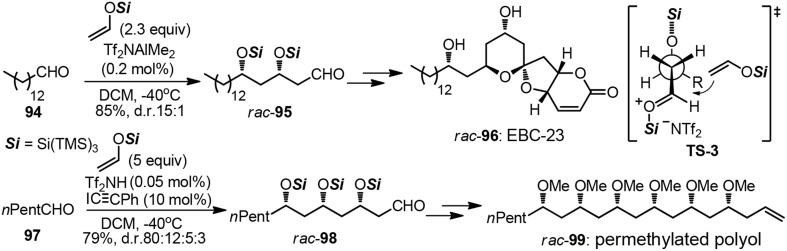
**Synthesis of *rac*-EBC-23 and *rac*-permethylated polyol (Albert and Yamamoto, 2011)**.

The strategic use of aldol chemistry plays a fundamental role in efficient assembly of variant polyoxygenated targets, particularly those of polyketide origin (Mahrwald, 2013). When it comes to the construction of polyacetate unit by iterative acetate aldol reactions, very few examples have been advanced to date. Although the above mentioned tactic may be in principle more closely related to biosynthetic origin, great challenges have been posed on stereochemical controlling issues. One-pot polyaldol cascade reactions elegantly developed by Yamamoto group allowed for the rapid access to natural products such as anticancer compound EBC-23 (**96**) and polymethoxy-1-alkene (**99**, Figure 12) (Albert and Yamamoto, 2010, 2011). In each case, aldol adducts **95** and **98** were formed as already protected tris(trimethylsilyl)silyl ether with high 1,3-stereoselection. The rational of the high *syn*–selectivity as is depicted in **TS-3**, which is arising from the extreme bulkiness of the supersilyl group. An asymmetric version remains a formidable challenge so far.

Other noteworthy iterative methods for the construction of 1,3-polyols have been used in the total synthesis of polyrhacitides B. Polyrhacitides B was envisioned by Kirsch group to be assembled by alternate Pd-catalyzed asymmetric allylic esterification and chain extension (Menz and Kirsch, 2009). Mohapatra and coworkers completed the synthesis of polyrhacitides B based on the iterative usage of Maruoaka allylation and iodocyclization (Mohapatra et al., 2014).

## Polypropionates

In a prototypical polypropionate chain, vicinal Me-OH carrying chiral centers are densely packed. The challenge of making 2-methyl-1,3-polyol array assemblage in laboratory arises from rapid access to enantiomerically pure products of enormous stereochemical diversity. Before aldol and related carbonyl addition reactions were privileged, stereocontrol in loose open chain systems have been achieved via rigid ring templates. In Woodward's masterful synthesis of erythromycin (Woodward et al., 1981), the repeated tactical use of *cis* fused dithiadecalin building block enabled diastereoselective formation of eight chiral centers found on the macrocylic aglycon. After Woodward's landmark work, great achievement has also been made in using thiopyran as template to access polypropionates in the past decades (Ward, 2011). Different from Woodward's strategy, Stork and Rychnovsky (1987) made use of an iterative butenolide construction of polypropionate chains to synthesize (+)-(9*S*)-dihydroerythronolide A.

**Figure 13:**
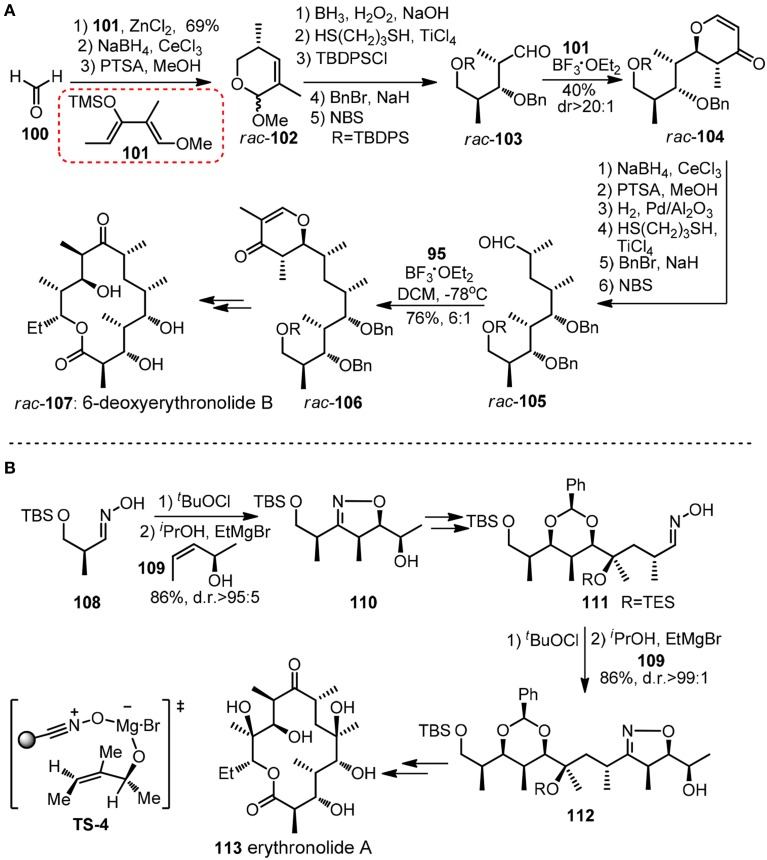
**(A)** Synthesis of *rac*-6-deoxyerythronolide B (Myles and Danishefsky, 1990); **(B)** Synthesis of erythronolide A (Bode et al., 2001).

Chiral pyranoid moieties may also serve as potential templates in acylic stereochontrol. Danishefsky and co-workers devised a synthesis of (±)-6-deoxyerythronolide (**107**, Figure 13A) featured by reiterative application of Lewis acid catalyzed cycloaddition of diene and aldehyde to stereoselectively generate *trans*-disubstituted dihydropyrone (Myles and Danishefsky, 1990). Danishefsky diene **101** and other dimethylated diene analogs equipped with exploitable functionality can be viewed as versatile dipropionyl building blocks. Following the same logic, polypropionate units in (+)-zincophorin and rifamycin S (**120**) have been conquered (Danishefsky and Selnick, 1987). Apart form those cycloaddition methods, an unique iterative sigmatropic strategy was used by Cywin and Kallmerten (1993) in their synthesis of C1-C11 subunit of zincophorin.

Besides Hetero Diels-Alder reaction, another iterative cycloaddition-based approach in polypropionate construction was disclosed by Carreira group (Bode et al., 2001). Their erythronolide A (**113**, Figure 13B) synthesis commenced with Kanemasa's Mg-mediated cycloaddtion between **108**-derived nitrile oxide and chiral allylic alcohol **109** to give alcohol **110**, whose isoxazoline unit served as a template to ensure a high diastereo-bias in subsequent tertiary hydroxy group formation. A repeated succession of **109** provided **111** as a masked β-hydroxyketone.

**Figure 14:**
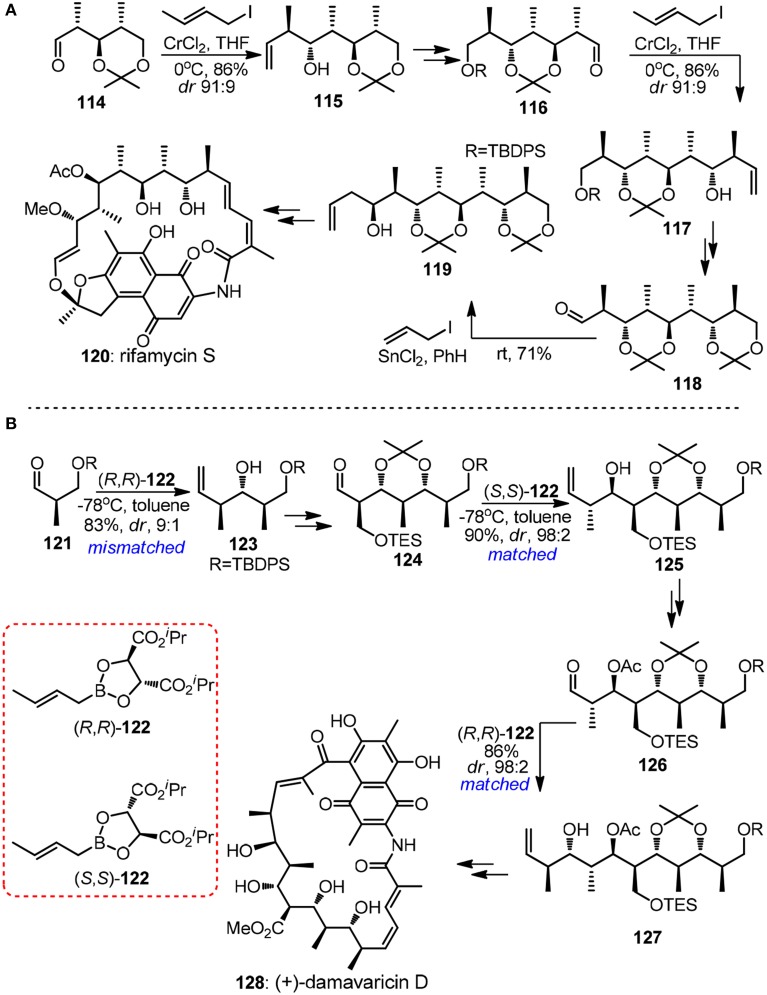
**(A)** Synthesis of rifamycin S (Nagaoka and Kishi, 1981); **(B)** Synthesis of (+)-damavaricin D (Roush et al., 1997).

The crotylmetal-aldehyde addition reaction has proven to be an universal approach for the modular preparation of polypropionate-derived natural products. Homoallylic alcohols of different stereodyads can be accessed and homologated aldehyde can be unveiled from its latent alkene functionality. The power of substrate-controlled crotylation was illustrated by Kishi's synthesis of aliphatic segment of ansamycin-type antibiotic rifamysin S (**120**, Figure 14A) (Nagaoka and Kishi, 1981). The Nozaki-Hiyama crotylchromium reagent, *in situ* prepared from either *trans*- or *cis*-crotyl iodide, smoothly reacted with α-methyl chiral aldehyde **114** to yield Felkin-adduct **115**. Although the substrate-directed approach may be attractive in terms of exceptionally low cost of crotylating reagent, its drawback is also apparent since its inability to realize diversity-oriented synthesis. Recently, progress has been made in the synthesis of 1,3,5-triols with iterative Cr-mediated catalytic asymmetric allylation reactions (Zhang et al., 2008).

The (+)-damavaricin D (**128**, Figure 14B) campaign in Roush's laboratory was marked with three highly reagent-controlled crotylations (Roush et al., 1997). Mismatched double asymmetric reaction between **123** and boronate (*R*,*R*)-**122**, which generated an desired *anti, anti*-stereotriad, was strategically designed to conduct at the very beginning of the whole synthetic sequence in order to obtain useful levels of diastereoselection. For advanced aldehyde intermediates **124** and **126** where overwhelming diastereofacial bias were expected as a result of the existence of more sterically demanding α-substituents, matched crotylation proceeded with exhibited excellent selectivity. Grée and co-workers (Ruiz et al., 2015) had demonstrated a new iterative tandem isomerization-aldolization sequence, where α-hydroxyallylsilanes can be viewed as propionaldehyde enolates equivalents. Aldehydes were generated from corresponding protected β-hydroxyacylsilanes via either photolytic cleavage or hydrogenolysis. Stereotetrads with *syn*-*anti* derivatives were obtained with only moderate stereoselectivity, which originates from preferentially *anti*-Felkin addition to chiral aldehyde.

The past three decades have witnessed the marvelous potentialities of aldol chemistry in assembling of complex polyoxygenated natural products as well as non-natural analogs. However, one of the most popular strategies involves a late-stage coupling of several advanced appropriately matched non-racemic fragments. As a laboratory emulation of nature's genius in polyketide biosynthesis, the iterative, monodirectional logic paves the way for rapid streamlined access of structural diversity through fine-tuning of the stereochemical nature of each aldolisations. Pioneering explorations in developing mono- and dipropionate synthones of various kinds had been led by Paterson and Scott (1999), Evans, etc. (Mahrwald, 2013).

**Figure 15:**
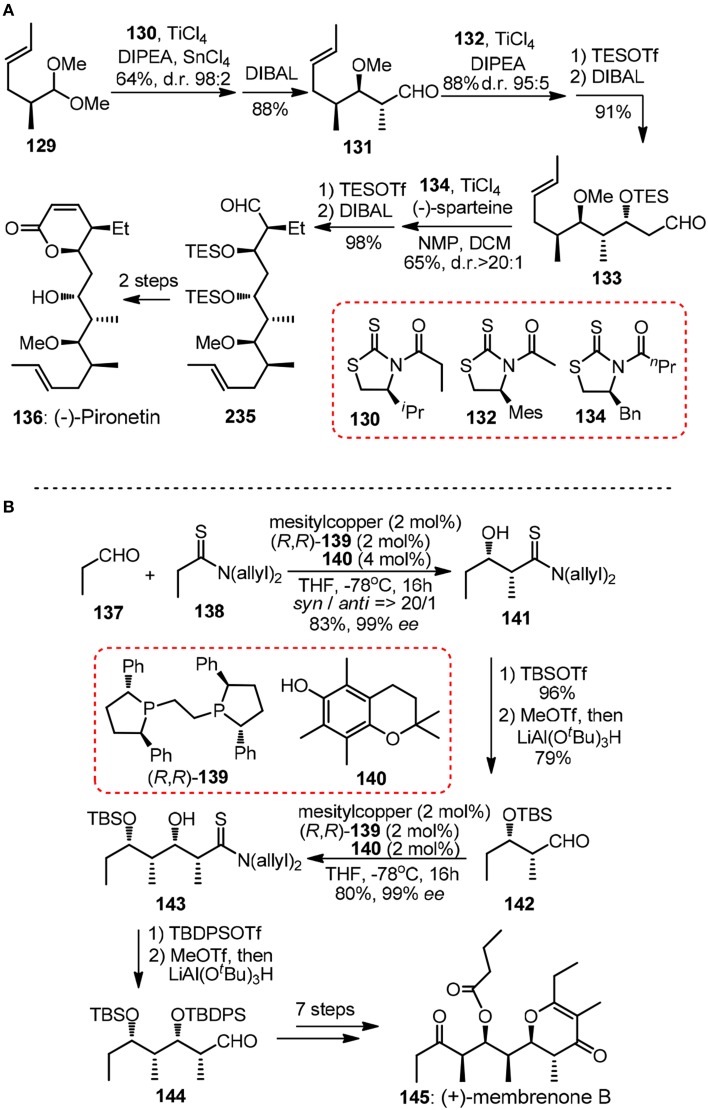
**(A)** Synthesis of (−)-pironetin (Crimmins and Dechert, 2009); **(B)** Synthesis of (+)-membrenone B (Alagiri et al., 2014).

Crimmins and Slade (2006) reported a formal synthesis of 6-deoxyerythronolide **107** validating the power of *N*-acylthiazolidinethiones as building blocks in consecutive aldol reactions. After that, a short enantioselective synthesis of (−)-pironetin (**136**, Figure 15A) was showcased (Crimmins and Dechert, 2009). The strictly linear sequence consisted of three highly selective auxiliary-based aldolisations including Urpi *anti* acetal aldol of chiral dimethylacetal **129**, acetal aldol using chlorotitanium enolate derived from mesityl-substituted **132** and a final *syn*–aldol. Note in each chain elongation cycle the reductive removal of the chiral thiazolidinethione is high yielding and straightforward, thus rendering the whole iteration particularly attractive.

Iterative application of catalytic asymmetric reactions in polypropionate construction has also been evolved by Shibasaki group in their synthesis of marine natural product (+)-membrenone B (**145**, Figure 15B) (Alagiri et al., 2014). Chemoselective enolization of thiopropionamide **138** proceeded catalytically under a soft Lewis acid/hard Brønsted base cooperative system. Aldehydes **142** and **144** can be delivered by facile reduction of more electrophilic iminium thioesters derived from silylated thioamide adducts.

**Figure 16:**
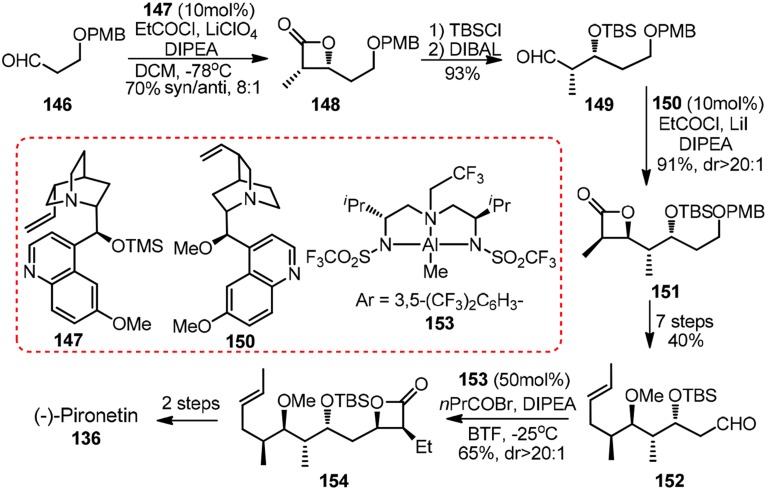
**Synthesis of (−)-pironetin (Shen et al., 2006)**.

Other noteworthy synthetic effort toward **136** came from Nelson and co-workers (Shen et al., 2006). Their featured asymmetric acyl halide-aldehyde cyclocondensations can be catalyzed by either cinchona alkaloid derived **147** and **150** or *in situ* generated aluminum complex **153** (Figure 16). A similar strategy for 1,3-polyols, in which iterative [2 + 2] asymmetric cycloaddition reaction of ketenes to *O*-protected α-hydroxy imines was involved, was developed by Palomo et al. (1993).

**Figure 17:**
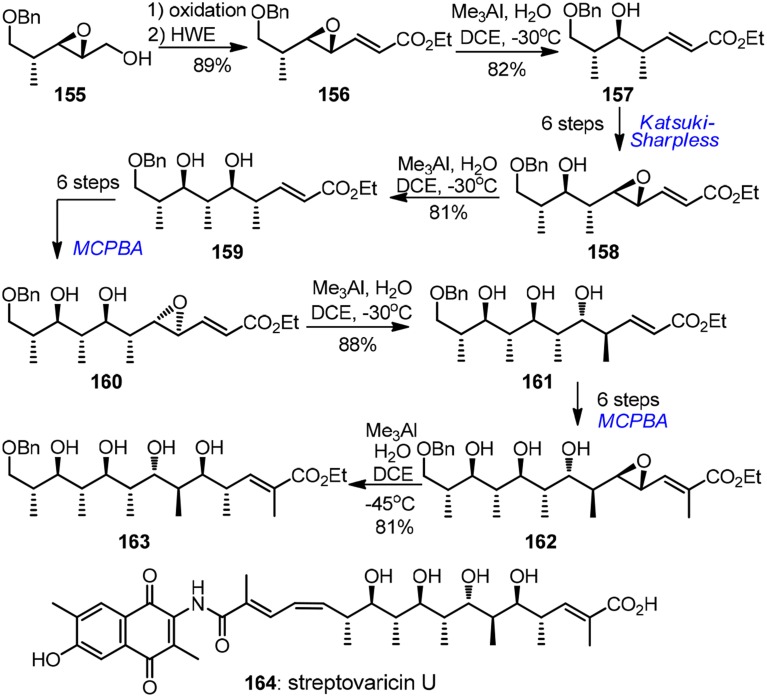
**Synthesis of streptovaricin U (Miyashita et al., 1991)**.

Apart from the above mentioned carbonyl chemistry-based approach, a stereoselective chiral epoxide ring-opening strategy has also been elaborated by several synthetic groups, albeit to a lesser extent. Excellent attempts in this area were conbtributed by Kishi (Corey and Hase, 1979; Nagaoka et al., 1980; Bartlett et al., 1982; Lipshutz and Kozlowski, 1984; Miyashita et al., 1991) and Shimizu (Oshima et al., 1989). An impressive example of alkenyloxirane methylation was disclosed by Miyashita's lab (1991) in their reiterative entry to the polypropionate ansa chain of antibiotic streptovaricin U (**164**, Figure 17). The key methylation reactions of γ,δ-epoxy acrylates **156**, **158**, **160**, and **162** occurred regioselectively at γ-position stereoselectively with net inversion of configuration. Chiral epoxy groups during each intervening homologation were introduced by Katsuki-Sharpless asymmetric epoxidation or substrate-controlled epoxidation. Water was found to be crucial to achieve the desired transformation. The major drawback, however, was that tedious protection group manipulations (not shown in Figure 17) were required. Triethylsilyl protection was necessary in the chain extension, but methylation of the corresponding polysilyloxy substrates turned out to be extremely sluggish. Additionally, chiral α,γ-dimethyl-γ,δ-epoxy acrylates were also developed to serve as propionate building blocks by Shimizu and co-workers (Oshima et al., 1989). The key epoxide ring-opening was realized by a stereoselective and regioselective Pd-catalyzed intramolecular hydride delivery. This methodology was further explored by Pujari and Kaliappan (2012) in their recent synthetic endeavor targeting all *syn* stereopentad framework of callystatin A.

The iterative strategies have also been successfully used in the total synthesis of polycyclic ethers (Zakarian et al., 2003; Kadota and Yamamoto, 2005; Clark, 2006). Though there are still many problems for an ideal automation synthesis, purification constitutes one of the tough ones. Recently, flow chemistry (Newton et al., 2014) and solid-phase synthesis (Umarye et al., 2006; Lee et al., 2007) were applied to the iterative synthesis of natural products and their derivatives.

## Future directions

In Reinhard W. Hoffmann's seminar review (Hoffmann, 1987) on the stereoselective syntheses of stereotrids as important precursors of polyketide natural products, he declared “*at first surprising, preferences for linear synthesis strategies is, in principle, the possibility of using an iterative procedure, which is amenable to automatization… However, reliable procedures for the specific construction of the stereotriads A to D are first required*.” It is clear that the intrinsic modular nature of diversified architectures found in complex polyketide natural products offers an ideal forum to demonstrate the salient utility of iterative strategies. Contrast to traditional laboratory synthesis where *personal preference* for designing synthetic plan to access different modules are uniquely designed, iterative synthesis facilitates synthetic effort by using similar reactants and well-developed conventional reaction conditions, thus holds considerable promise for rapid streamlined assembly of the entire spectrum of functionality and stereochemical permutations as will. Several elegant examples have been illustrated in the synthesis of catechin oligomers by Suzuki and co-workers (Ohmori et al., 2011) and McDonald's interative synthesis of poly-deoxysugers based on the alkynol cycloisomerization (McDonald and Zhu, 1998). Despite the fact that the emergence of iterative design in polyketide synthesis over the last few decades has been rather dramatic, a number of gaps have remained. Later comprise in terms of the stoichiometric use of chiral controlling reagents, tediously lengthy protocols in building block preparation as well as intervening *re*-functionalization operations, repeated use of protecting groups to suppress uncontrolled polymerization as well as other side reactions, the elusive and intractable stereo-controlling nature of the crucial reactions that used in a reiterative fashion. Nevertheless, these problems seem to cast light on the challenges the synthetic communities have to cope with in developing an ideal biomimetic iteration strategy. The robustness of the strategy will enabled more spectacular synthetic accomplishments in the coming future.

### Conflict of interest statement

The authors declare that the research was conducted in the absence of any commercial or financial relationships that could be construed as a potential conflict of interest.

## References

[B1] Abiko A. Masamune S. (1996). Synthesis of (+)-siphonarienone: asymmetric alkylation using a chiral benzopyrano-isoxazolidine auxiliary. Tetrahedron Lett. 37, 1081–1083. 10.1016/0040-4039(95)02353-4

[B2] Alagiri K. Lin S.-Q. Kumagai N. Shibasaki M. (2014). Iterative direct aldol strategy for polypropionates: enantioselective total synthesis of (−)-membrenone A and B. Org. Lett. 16, 5301–5303. 10.1021/ol5024932 25259628

[B3] Albert B. J. Yamamoto H. (2010). A triple-aldol cascade reaction for the rapid assembly of polyketides. Angew. Chem. Int. Ed. 49, 2747–2749. 10.1002/anie.200907076 20229553 PMC3066012

[B4] Albert B. J. Yamamoto H. (2011). Rapid total syntheses utilizing “supersilyl” chemistry. Angew. Chem. Int. Ed. 50, 2610–2612. 10.1002/anie.201007210 21370348 PMC3086072

[B5] Balieu S. Hallett G. E. Burns M. Bootwicha T. Studley J. Aggarwal V. K. (2015). Toward ideality: the synthesis of (+)-kalkitoxin and (+)-hydroxyphthioceranic acid by assembly-line synthesis. J. Am. Chem. Soc. 137, 4398–4403. 10.1021/ja512875g 25625684

[B6] Bartlett P. A. Meadows J. D. Brown E. G. Morimoto A. Junstedt K. K. (1982). Carbonate extension. A versatile procedure for functionalization of acyclic homoallylic alcohols with moderate stereocontrol. J. Org. Chem. 47, 4013–4018. 10.1021/jo00142a002

[B7] Blair D. J. Fletcher C. J. Wheelhouse K. Atherine M. P. Aggarwal V. K. (2014). Stereocontrolled synthesis of adjacent acyclic quaternary-tertiary motifs: application to a concise total synthesis of (−)-filiformin. Angew. Chem. Int. Ed. 53, 5552–5555. 10.1002/anie.201400944 24757079

[B8] Bode J. W. Fraefel N. Muri D. Carreira E. M. (2001). A general solution to the modular synthesis of polyketide building blocks by Kanemasa hydroxy-directed nitrile oxide cycloadditions. Angew. Chem. Int. Ed. 40, 2082–2085. 10.1002/1521-3773(20010601)40:11<2082::AID-ANIE2082>3.0.CO;2-1 29712193

[B9] Bode S. E. Wolberg M. Müller M. (2006). Stereoselective synthesis of 1,3-diols. Synthesis 557–588. 10.1055/s-2006-926315

[B10] BouzBouz S. Cossy J. (2003). Total synthesis of (+)-strictifolione. Org. Lett. 5, 1995–1997. 10.1021/ol034619s 12762705

[B11] Burns M. Essafi S. Bame J. R. Bull S. P. Matthew P. Balieu S.. (2014). Assembly-line synthesis of organic molecules with tailored shapes. Nature 513, 183–188. 10.1038/nature13711 25209797 PMC4167605

[B12] Caruthers M. H. (1985). Gene synthesis machines: DNA chemistry and its uses. Science 230, 281–285. 10.1126/science.3863253 3863253

[B13] Clark J. S. (2006). Construction of fused polycyclic ethers by strategies involving ring-closing metathesis. Chem. Commun. 3571–3581. 10.1039/b601839d 17047770

[B14] Corey E. J. Hase T. (1979). Studies on the total synthesis of rifamycin. Highly stereoselective synthesis of intermediates for construction of the C(15) to C(29) chain. Tetrahedron Lett. 20, 335–338. 10.1016/S0040-4039(01)85964-5

[B15] Crimmins M. T. Dechert A.-M. R. (2009). Enantioselective total synthesis of (−)-pironetin: iterative aldol reactions of thiazolidinethiones. Org. Lett. 11, 1635–1638. 10.1021/ol9003228 19281219 PMC2701212

[B16] Crimmins M. T. Slade D. J. (2006). Formal synthesis of 6-deoxyerythronolide B. Org. Lett. 8, 2191–2194. 10.1021/ol0607241 16671814 PMC2546576

[B17] Cywin C. L. Kallmerten J. (1993). Synthetic studies of the ionophore antibiotic zincophorin. 2. Iterative sigmatropic construction of the C1-C11 subunit. Tetrahedron Lett. 34, 1103–1106. 10.1016/S0040-4039(00)77501-0

[B18] Danishefsky S. J. Selnick H. G. (1987). Total synthesis of zincophorin. J. Am. Chem. Soc. 109, 1572–1574. 10.1021/ja00239a049

[B19] Dutta S. Whicher J. R. Hansen D. A. Hale W. A. Chemler J. A. Congdon G. R.. (2014). Structure of a modular polyketide synthase. Nature 510, 512–517. 10.1038/nature13423 24965652 PMC4278352

[B20] Evans P. A. Grisin A. Lawler M. J. (2012). Diastereoselective construction of *syn*-1,3-dioxanes via a Bismuth-mediated two-component hemiacetal/oxa-conjugate addition reaction. J. Am. Chem. Soc. 132, 15559–15561. 10.1021/ja208668u 22296255

[B21] Feuerbacher N. Vögtle F. (1998). Iterative, in Dendrimers, ed Vögtle F. (Berlin; Heidelberg: Springer), 1–18.

[B22] Garcia-Fortanet J. Murga J. Carda M. Alberto J. (2003). On the structure of passifloricin A: asymmetric synthesis of the α-lactones of (2*Z*, 5*S*, 7*R*, 9*S*, 11*S*)- and (2*Z*, 5*R*, 7*R*, 9*S*, 11*S*)-tetrahydroxyhexacos-2-enoic Acid. Org. Lett. 5, 1447–1449. 10.1021/ol034182o 12713295

[B23] Geerdink D. Horst T. B. Lepore M. Mori L. Puzo G. Hirsch A. K. (2013). Total synthesis, stereochemical elucidation and biological evaluation of Ac2SGL; a 1,3-methyl branched sulfoglycolipid from Mycobacterium tuberculosis. Chem. Sci. 4, 709–716. 10.1039/c2sc21620e

[B24] Ghosh A. K. Liu C. (2001). Total synthesis of antitumor depsipeptide (−)-doliculide. Org. Lett. 3, 635–638. 10.1021/ol0100069 11178844

[B25] Gillis E. P. Burke M. D. (2007). A simple and modular strategy for small molecule synthesis: iterative Suzuki-Miyaura coupling of B-protected haloboronic acid building blocks. J. Am. Chem. Soc. 129, 6716–6717. 10.1021/ja0716204 17488084

[B26] Gillis E. P. Burke M. D. (2009). Iterative cross-couplng with MIDA boronates: towards a general platform for small molecule synthesis. Aldrichimica Acta. 42, 17–27. 22523433 PMC3328809

[B27] Han S. B. Hassan A. Kim I. S. Krische M. J. (2010). Total synthesis of (+)-roxaticin via C-C bond forming transfer hydrogenation: a departure from stoichiometric chiral reagents, auxiliaries, and premetalated nucleophiles in polyketide construction. J. Am. Chem. Soc. 132, 15559–15561. 10.1021/ja1082798 20961111 PMC2975273

[B28] Hanessian S. Giroux S. Mascitti V. (2006). The iterative synthesis of acyclic deoxypropionate units and their implication in polyketide-derived natural products. Synthesis 1057–1076. 10.1055/s-2006-926376

[B29] Hoffmann R. W. (1987). Stereoselective syntheses of building blocks with three consecutive stereogenic centers: important precursors of polyketide natural products. Angew. Chem. Int. Ed. 26, 489–503. 10.1002/anie.198704893

[B30] Hoffmann R. W. (2000). Conformation design of open chain compounds. Angew. Chem. Int. Ed. 39, 2054–2070. 10.1002/1521-3773(20000616)39:12<2054::AID-ANIE2054>3.0.CO;2-Z 10941017

[B31] Horst B. T. Feringa B. L. Minnaard A. J. (2010). Iterative strategies for the synthesis of deoxypropionates. Chem. Commun. 46, 2535–2547. 10.1039/B926265B 20449305

[B32] Kadota I. Yamamoto Y. (2005). Synthetic strategies of marine polycyclic ethers via intramolecular allylations: linear and convergent approaches. Acc. Chem. Res. 38, 423–432. 10.1021/ar040118a 15895980

[B33] Katsuki T. Lee A. W. Ma M. P. Martin V. S. Masamune S. Sharpless K. B. (1982). Synthesis of saccharides and related polyhydroxylated natural products. 1. Simple Alditols. J. Org. Chem. 47, 1373–1378. 10.1021/jo00346a051

[B34] Katz L. (1997). Manipulation of modular polyketide synthases. Chem. Rev. 97, 2557–2575. 10.1021/cr960025+ 11851471

[B35] Kim I. S. Ngai M.-Y. Krische M. J. (2008). Enantioselective Iridium-catalyzed carbonyl allylation from the alcohol or aldehyde oxidation level using allyl acetate as an allyl metal surrogate. J. Am. Chem. Soc. 130, 6340–6341. 10.1021/ja802001b 18444616 PMC2858451

[B36] Kumar P. Pandey M. Guptaa P. Dhavale D. D. (2012). Organocatalytic stereoselective synthesis of passifloricin A. Org. Biomol. Chem. 10, 1820–1825. 10.1039/c2ob06711k 22246108

[B37] Lee S. J. Anderson T. M. Burke M. D. (2010). A simple and general platform for generating stereochemically complex polyene frameworks by iterative cross-coupling. Angew. Chem. Int. Ed. 49, 8860–8863. 10.1002/anie.201004911 PMC3037596 20927795

[B38] Lee S. Lee T. Lee Y. Kim D. Kim S. (2007). Solid-phase library synthesis of polyynes similar to naturalproducts. Angew. Chem. Int. Ed. 46, 8422–8425. 10.1002/anie.200703208 17893942

[B39] Leonori D. Aggarwal V. K. (2014). Lithiation-borylation methodology and its application in synthesis. Acc. Chem. Res. 47, 3174–3183. 10.1021/ar5002473 25262745

[B40] Li J. Ballmer S. G. Gillis E. P. Fujii S. Schmidt M. J. Palazzolo A. M.. (2015). Synthesis of many different types of organic small molecules using one automated process. Science 347, 1221–1226. 10.1126/science.aaa5414 25766227 PMC4687482

[B41] Lipshutz B. H. Kozlowski J. A. (1984). A reiterative route to chiral all-*syn*-1,3-polyols. J. Org. Chem. 49, 1147–1149. 10.1021/jo00180a045

[B42] Magnin-Lachaux M. Tan Z. Liang B. Negishi E. (2004). Efficient and selective synthesis of siphonarienolone and related reduced polypropionates via Zr-catalyzed asymmetric carboalumination. Org. Lett. 6, 1425–1427. 10.1021/ol0497483 15101758

[B43] Mahrwald R. (2013). Modern Methods in Stereoselective Aldol Reactions. Weinheim: Wiley-VCH Verlag GmbH & Co. KGaA.

[B44] Matteson D. S. (2013). Boronic esters in asymmetric synthesis. J. Org. Chem. 78, 10009-10023. 10.1021/jo4013942 23875690

[B45] McDonald F. E. Zhu H. Y. H. (1998). Novel strategy for oligosaccharide synthesis featuring reiterative alkynol cycloisomerization. J. Am. Chem. Soc. 120, 4246–4247. 10.1021/ja980196r

[B46] Menz H. Kirsch S. F. (2009). Total synthesis of polyrhacitides A and B by use of an iterative strategy for the stereoselective synthesis of 1,3-polyol arrays. Org. Lett. 14, 5634–5637. 10.1021/ol902135v 19919037

[B47] Merrifield R. B. (1985). Solid phase synthesis (Nobel Lecture). Angew. Chem. Int. Ed. 24, 799–810. 10.1002/anie.198507993

[B48] Miyashita M. Hoshino M. Yoshikoshi A. (1991). Stereospecific methylation of.gamma., delta.-epoxy acrylates by trimethylaluminum: a method for the iterative construction of polypropionate chains. J. Org. Chem. 56, 6483–6485. 10.1021/jo00023a001

[B49] Mohapatra D. K. Krishnarao P. S. Bhimireddy E. Yadav J. S. (2014). Iterative iodocyclization: total synthesis of polyrhacitide B. Synthesis 46, 1639–1647. 10.1055/s-0033-1341154

[B50] Myles D. C. Danishefsky S. J. (1990). Development of a fully synthetic stereoselective route to 6-deoxyerythronolide B by reiterative applications of the Lewis acid catalyzed diene aldehyde cyclocondensation reaction: a remarkable instance of diastereofacial selectivity. J. Org. Chem. 55, 1636–1648. 10.1021/jo00292a045

[B51] Nagaoka H. Kishi Y. (1981). Further synthetic studies on rifamycin S. Tetrahedron 37, 3873–3888.

[B52] Nagaoka H. Rutsch W. Schimid G. Lio H. Johnson M. R. Kishi Y. (1980). Total synthesis of rifamycins. 1. stereocontrolled synthesis of the aliphatic building block. J. Am. Chem. Soc. 102, 7962–7965. 10.1021/ja00547a037

[B53] Newton S. Carter C. F. Pearson C. M. Alves L. de C. Lange H. (2014). Accelerating spirocyclic polyketide synthesis using flow chemistry. Angew. Chem. Int. Ed. 53, 4915–4920. 10.1002/anie.201402056 24729438

[B54] Nicolaou K. C., R. Daines A. Chakraborty T. K. Ogawa Y. (1987). Total synthesis of amphotericin B. J. Am. Chem. Soc. 109, 2821–2822. 10.1021/ja00243a043

[B55] Ohmori K. Shono T. Hatakoshi Y. Yano T. Suzuki K. (2011). Integrated synthetic strategy for higher catechin oligomers. Angew. Chem. Int. Ed. 50, 4862–4867. 10.1002/anie.201007473 21472924

[B56] Oshima M. Yamazaki H. Shimizu I. Nisar M. Tsuji J. (1989). Palladium-catalyzed selective hydrogenolysis of alkenyloxiranes with formic acid. Stereoselectivity and synthetic utility. J. Am. Chem. Soc. 111, 6280–6287. 10.1021/ja00198a045

[B57] Palomo C. Aizpurua J. M. Urchegui R. Garcfa J. M. (1993). A new entry to 1,3-Polyols, 2-amino 1,3-polyols, and beta-(l-hydroxyalkyl)isoserines using azetidinone frameworks as chiral templates via iterative asymmetric [2 + 2] cycloaddition reactions. J. Org. Chem. 58, 1646–1648. 10.1021/jo00059a003

[B58] Paterson I. Scott J. P. (1999). Laboratory emulation of polyketide biosynthesis: an iterative, aldol-based, synthetic entry to polyketide libraries using (R)- and (S)-1-(benzyloxy)-2-methylpentan-3-one, and conformational aspects of extended polypropionates. J. Chem. Soc. Perkin Trans. 1, 1003–1014. 10.1039/A809818B

[B59] Pujari S. A. Kaliappan K. P. (2012). An iterative Shimizu non-aldol approach for the stereoselective synthesis of C13-C22 fragment of callystatin A. Org. Biomol. Chem. 10, 1750–1753. 10.1039/c2ob06838a 22286400

[B60] Rasappan R. Aggarwal V. K. (2014). Synthesis of hydroxyphthioceranic acid using a traceless lithiation–borylation–protodeboronation strategy. Nat. Chem. 6, 810–814. 10.1038/nchem.2010 25143217

[B61] Roush W. R. Coffey D. S. Madar D. J. (1997). Total synthesis of (+)-damavaricin D. J. Am. Chem. Soc. 119, 11331–11332. 10.1021/ja971828x

[B62] Ruiz J. Murthy A. S. Roisnel T. Chandrasekhar S. Grée R. (2015). α-Hydroxyallylsilanes as propionaldehyde enolate equivalents and their use toward iterative aldol reactions. J. Org. Chem. 80, 2364–2375. 10.1021/acs.joc.5b00031 25636066

[B63] Rychnovsky S. D. Griesgraber G. (1992). An iterative and convergent synthesis of *syn* polyols. J. Org. Chem. 57, 1559–1563. 10.1021/jo00031a041

[B64] Seeberger P. H. Haase W.-C. (2000). Solid-phase oligosaccharide synthesis and combinatorial carbohydrate libraries. Chem. Rev. 100, 4349–4393. 10.1021/cr9903104 11749351

[B65] Shen X. Wasmuth A. S. Zhao J. Zhu C. Nelson S. G. (2006). Catalytic asymmetric assembly of stereodefined propionate units: an enantioselective total synthesis of (−)-pironetin. J. Am. Chem. Soc. 128, 7438–7439. 10.1021/ja061938g 16756287

[B66] Staunton J. Weissman K. J. (2001). Polyketide biosynthesis: a millennium review. Nat. Prod. Rep. 18, 380–416. 10.1039/a909079g 11548049

[B67] Stork G. Rychnovsky S. D. (1987). Iterative butenolide construction of polypropionate chains. Application to an efficient synthesis of (+)(9*S*)-dihydroerythronolide A. Pure Appl. Chem. 59, 345–352. 10.1351/pac198759030345

[B68] Tosaki S.-Y. Horiuchi Y. Nemoto T. Ohshima T. Shibasaki M. (2004). Strategy for enantio- and diastereoselective syntheses of all possible stereoisomers of 1,3-Polyol arrays based on a highly catalyst-controlled epoxidation of α,β-unsaturated morpholinyl amides: application to natural product synthesis. Chem. Eur. J. 10, 1527–1544. 10.1002/chem.200305709 15034897

[B69] Umarye J. D. Leßmann T. García A. B. Mamane V. Sommer S. Waldmann H. (2006). Biology-oriented synthesis of stereochemically diverse natural-product-derived compound collections by iterative allylations on a solid support. Chem. Eur. J. 13, 3305–3319. 10.1002/chem.200601698 17310497

[B70] Wang C. Glorius F. (2008). Controlled iterative cross-coupling: on the way to the automation of organic synthesis. Angew. Chem. Int. Ed. 48, 5240–4244. 10.1002/anie.200901680 19479924

[B71] Ward D. E. (2011). The thiopyran route to polypropionates. Chem. Commun. 47, 11375–11393. 10.1039/c1cc13323c 21761054

[B72] Whicher J. R. Dutta S. Hansen D. A. Hale W. A. Chemler J. A. Dosey A. M.. (2014). Structural rearrangements of a polyketide synthase module during its catalytic cycle. Nature 510, 560–564. 10.1038/nature13409 24965656 PMC4074775

[B73] Woerly E. M. Roy J. Burke M. D. (2014). Synthesis of most polyene natural product motifs using just 12 building blocks and one coupling reaction. Nat. Chem. 6, 484–491. 10.1038/NCHEM.1947 24848233 PMC4079739

[B74] Woodward R. B. Logusch E. Nambiar K. P. Sakan K. Ward D. E. Au-Yeung B. W. (1981). Asymmetric total synthesis of erythromycin. 2. Synthesis of an erythronolide a lactone system. J. Am. Chem. Soc. 103, 3210–3213. 10.1021/ja00401a050

[B75] Yadav J. S. Rao P. P. Reddy M. S. Rao N. V. Prasad A. R. (2007). Stereoselective synthesis of (+)-cryptocarya diacetate by an iterative Prins cyclisation and reductive cleavage sequence. Tetrahedron Lett. 48, 1469–1471. 10.1016/j.tetlet.2006.12.068

[B76] Zakarian A. Batch A. Holton R. A. (2003). A convergent total synthesis of hemibrevetoxin B. J. Am. Chem. Soc. 26, 7822–7824. 10.1021/ja029225v 12822999

[B77] Zhang Z. Aubry S. Kishi Y. (2008). Iterative Cr-mediated catalytic asymmetric allylation to synthesize *syn*/*syn*- and *anti*/*anti*-1,3,5-triols. Org. Lett. 10, 3077–3080. 10.1021/ol801094e 18549227

